# Investigating the research trajectory and future trends in type 2 diabetes mellitus and aging: a bibliometric analysis from 2009 to 2025 based on big data

**DOI:** 10.3389/fragi.2026.1779773

**Published:** 2026-04-22

**Authors:** Tingting Gong, Wei Jiang, Yiman Han, Yang Liu, Wuhui Zhuo, Shengyao Li, Zhipeng Hu, Zixi Zeng, Rensong Yue, Maoyi Yang

**Affiliations:** Hospital of Chengdu University of Traditional Chinese Medicine, Chengdu, China

**Keywords:** aging, bibliometric analysis, cellular senescence, research trends, senolytics, type 2 diabetes mellitus

## Abstract

**Background:**

The relationship between type 2 diabetes mellitus (T2DM) and aging has attracted growing scientific attention. Growing evidence suggests that T2DM is not only a metabolic disorder but also a condition associated with accelerated biological aging. This study aimed to systematically map the global research landscape, intellectual structure, and emerging trends at the intersection of T2DM and aging using bibliometric approaches.

**Methods:**

Publications indexed in the Web of Science Core Collection from 1 January 2009 to 31 December 2025 were retrieved, yielding 3,048 records. Bibliometric analyses were conducted using VOSviewer, CiteSpace, and R to construct collaboration networks, co-citation structures, and keyword evolution patterns.

**Results:**

After a moderate growth phase from 2011 to 2015, publication output increased markedly from 2016 onward and reached a peak in 2025, accompanied by the progressive formation of an international collaboration network dominated by the United States and China. Mechanistic studies constituted the primary research focus, particularly those related to cellular senescence, oxidative stress, and inflammation. Cellular senescence emerged as a structurally central node within the knowledge network. Thematic evolution analysis further revealed increasing attention to aging-related comorbidities, including cardiovascular disease, Alzheimer’s disease, erectile dysfunction, and cognitive impairment. Recent research fronts have increasingly focused on molecular pathways, including the senescence-associated secretory phenotype, the NLRP3 inflammasome, and epigenetic regulation.

**Conclusion:**

This bibliometric analysis provides a comprehensive overview of the evolving research landscape linking T2DM and aging. The prominence of senescence-related pathways highlights a growing convergence between diabetes research and aging biology. Emerging strategies targeting fundamental aging mechanisms—including senolytic therapies and glucose-lowering drugs with potential geroprotective effects such as metformin and empagliflozin—represent promising directions for future research.

## 1 Introduction

The conventional understanding of type 2 diabetes mellitus (T2DM) is increasingly being reconsidered. Accumulating evidence suggests that T2DM is not merely a metabolic disorder but may also exhibit characteristics of accelerated systemic aging (Wölfel and Kassem, 2025). This emerging perspective is consistent with the central tenets of geroscience, which propose that aging serves as a common upstream driver of most chronic diseases and that targeting its underlying biological mechanisms may enable coordinated prevention and management across multiple conditions (Kennedy et al., 2014; Xu et al., 2018).

Studies indicate that Phenotypic Age Acceleration (PhenoAgeAccel), a metric for assessing biological aging constructed from clinical chemistry biomarkers, is independently associated with the risk of T2DM onset, progression, and complications (Pan et al., 2025). Beyond hallmark metabolic abnormalities, individuals with T2DM frequently demonstrate premature, multisystem functional decline affecting the cardiovascular, cognitive, and renal systems (Crane et al., 2013; Chen et al., 2018; Ryder et al., 2020).

Accordingly, therapeutic strategies aimed at modulating aging-related mechanisms, including the clearance of senescent cells, have shown potential to ameliorate aging hallmarks while improving metabolic outcomes (Xu et al., 2018). Building upon established glycemic management strategies, these approaches may represent a complementary extension of current T2DM management paradigms.

With the rapid growth of publications in this interdisciplinary domain, the knowledge landscape of T2DM and aging research has become increasingly complex. Traditional narrative reviews may be insufficient to objectively and quantitatively characterize its developmental trajectory, collaboration patterns, and evolving research themes. Bibliometric analysis provides a systematic quantitative framework for mapping disciplinary evolution, research networks, and emerging trends through visualization techniques (Donthu et al., 2021).

To date, however, dedicated bibliometric investigations focusing on the intersection between T2DM and aging remain limited. This gap constrains a comprehensive understanding of the field’s intellectual landscape and research dynamics. To address this need, we conducted a systematic bibliometric analysis of relevant literature indexed in the Web of Science Core Collection (WoSCC) and published between 1 January 2009, and 31 December 2025. The present study aimed to map the global research landscape, identify major knowledge domains and evolutionary trajectories, and reveal emerging research frontiers in the intersection of T2DM and aging.

## 2 Materials and methods

### 2.1 Data source and search strategy

To ensure data consistency, citation stability, and compatibility with bibliometric software, the WoSCC was selected as the primary data source. WoSCC is widely recognized for its rigorous indexing standards and well-structured citation metadata, which are particularly suitable for co-citation and network analyses (Mongeon and Paul-Hus, 2016). The use of a single database also minimizes potential biases arising from duplicate records and heterogeneous metadata formats across databases, while ensuring comparability with previous bibliometric studies in this field.

The search strategy was designed to balance comprehensiveness and precision. Search terms were developed based on Medical Subject Headings (MeSH) and related entry terms to ensure comprehensive coverage of concepts related to T2DM and aging. Although WoSCC does not directly apply MeSH indexing, MeSH terminology was used as a controlled vocabulary framework to systematically develop topic search strings. Boolean operators were used to combine synonymous terms and thematic concepts.

The final search query was: TS= (“Type 2 Diabetes” OR “Diabetes Mellitus, Type 2” OR “NIDDM” OR “Non-Insulin-Dependent Diabetes Mellitus” OR “Diabetes Mellitus, Non-Insulin Dependent” OR “Diabetes Mellitus, Type II” OR “Diabetes Mellitus, Stable”) AND TS= (“Aging” OR “Senescence”). The time span was set from 1 January 2009, to 31 December 2025. Data were retrieved and downloaded from WoSCC on 13 February 2026.

### 2.2 Study selection and data extraction

Literature screening was conducted according to predefined inclusion and exclusion criteria. Eligible publications included original research articles and review articles focusing on the intersection between T2DM and aging, published in English between 1 January 2009, and 31 December 2025. Non-research publication types—including meeting abstracts, editorial materials, book chapters, proceeding papers, letters, corrections, news items, and retracted articles—were excluded. Duplicate records were removed through DOI matching and verification of WoS accession numbers (Page et al., 2021). Two investigators independently screened titles and abstracts. Discrepancies were resolved through discussion, with a third researcher serving as arbiter when necessary (Bramer et al., 2018).

**FIGURE 1:**
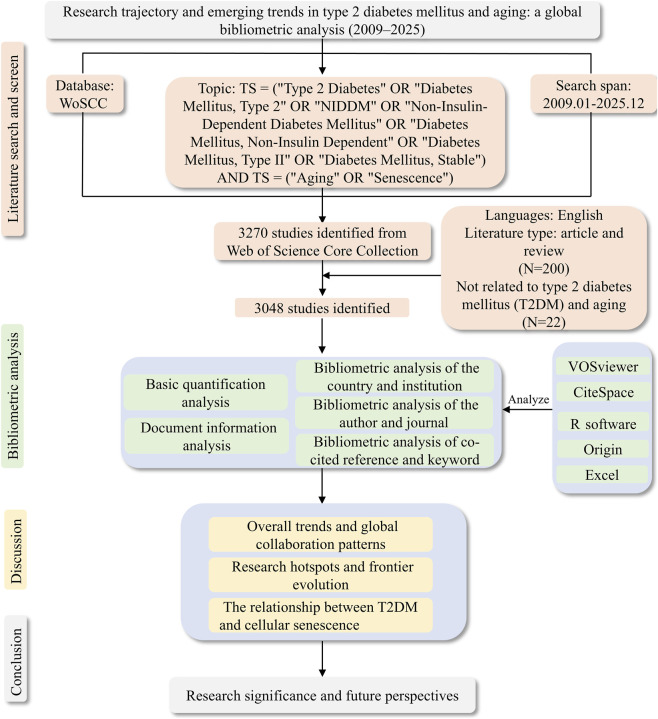
Flowchart of the study design, illustrating the data retrieval, screening, and bibliometric analysis procedures.

A total of 3,070 records were initially identified. After excluding 22 ineligible documents, 3,048 publications were included in the final analytical dataset (Figure 1). Bibliometric metadata—including titles, authors, affiliations, journals, keywords, abstracts, cited references, and publication years—were exported in plain text format from WoSCC for subsequent bibliometric analysis.

Author name disambiguation was performed through cross-verification of institutional affiliations, co-authorship patterns, and thematic consistency of publications. High-frequency authors and names with common transliterations were manually examined to minimize potential misattribution bias. Country or regional attribution followed the classification rules of WoSCC, based on authors’ institutional affiliations. Keyword normalization was conducted by merging synonyms, unifying singular and plural forms, and correcting spelling variations to enhance analytical precision (van Eck and Waltman, 2017).

### 2.3 Data analysis and visualization

Bibliometric analyses were conducted using VOSviewer (version 1.6.20), CiteSpace (version 6.4.R1), R (version 4.5.1), Microsoft Office Excel 2021, Origin 2024, and SCImago Graphica. Country collaboration networks were constructed using VOSviewer, with a minimum threshold of five publications per country. The geographical distribution of international collaborations was visualized using SCImago Graphica.

Co-cited author analysis, document co-citation analysis, and keyword co-occurrence analysis were conducted using CiteSpace. Time slicing was set from 2009 to 2025 with 1-year intervals. The g-index (k = 25) was applied as the node selection criterion. The Pathfinder pruning algorithm was used to simplify network structures and identify key intellectual bases and evolutionary trajectories. Data preprocessing and descriptive statistical analyses were performed in R. Metadata organization was assisted by Excel, and additional graphical visualizations were generated using Origin.

## 3 Results

### 3.1 Publication trends and disciplinary structure

**FIGURE 2:**
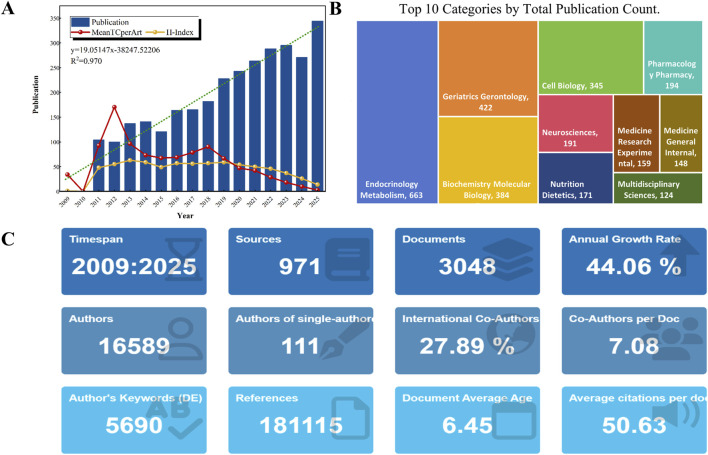
Research landscape of T2DM and aging (2009–2025). **(A)** Annual publication output, mean total citations per article, H-index, and fitted trend curve of publication counts. **(B)** Top 10 subject categories by publication volume. **(C)** Key bibliometric indicators derived from the R package *bibliometrix*.

A total of 3,048 publications between 1 January 2009 and 31 December 2025 were included. These studies were published in 971 peer-reviewed journals and involved 16,589 authors (Figure 2C). Figure 2A shows the annual publication output, H-index, mean total citations per article, and the fitted growth curve (y = 19.05147x − 38,247.52206, R^2^ = 0.970). Between 2009 and 2015, publication output increased steadily. A pronounced rise was observed beginning in 2016 (n = 164), with a peak reached in 2025 (n = 344), indicating sustained growth of research activity in this field.

Original research articles accounted for 2,102 publications (68.96%), whereas reviews comprised 946 publications (31.04%). The dataset yielded a mean citation rate of 50.63 citations per article, an international co-authorship rate of 27.89%, and an average of 7.08 co-authors per publication (Figure 2C). Subject category analysis (Figure 2B) shows that *Endocrinology & Metabolism* (21.75%) and *Geriatrics & Gerontology* (13.85%) are the two leading disciplines, together accounting for over one-third of the total output and underscoring the interdisciplinary structure of this research domain.

### 3.2 Analysis of national research output and collaboration

A total of 82 countries/regions contributed to research on T2DM and aging. The United States produced the largest number of publications (n = 780, 25.6%), followed by China (n = 578, 19.0%), Italy (n = 199, 6.5%), and Japan (n = 140, 4.6%) (Table 1). The United States recorded the highest number of multiple-country publications (MCP; n = 184), although its MCP ratio was 23.6%, whereas the United Kingdom showed a higher MCP proportion (45.3%).

**TABLE 1:** Top 10 countries/regions by number of publications in T2DM and aging research (2009–2025).

Rank	Country/Region	Articles	Percentage (%) (N = 3048)	SCP	MCP	MCP ratio (%)	TC	Average citations
1	USA	780	25.6	596	184	23.6	61,682	79.1
2	China	578	19.0	480	98	17.0	15,913	27.5
3	Italy	199	6.5	136	63	31.7	9,913	49.8
4	Japan	140	4.6	124	16	11.4	4,113	29.4
5	Germany	116	3.8	69	47	40.5	7,333	63.2
6	Spain	107	3.5	69	38	35.5	4,484	41.9
7	United Kingdom	95	3.1	52	43	45.3	5,557	58.5
8	Canada	93	3.1	55	38	40.9	4,887	52.5
9	South Korea	79	2.6	58	21	26.6	3,325	42.1
10	Brazil	73	2.4	53	20	27.4	1,776	24.3

TC, total citations; H-Index, quantifying both productivity and citation impact; SCP, single-country publications; MCP, multiple-country publications; MCP, Ratio = MCP/Articles. data were analyzed using *bibliometrix*.

**FIGURE 3:**
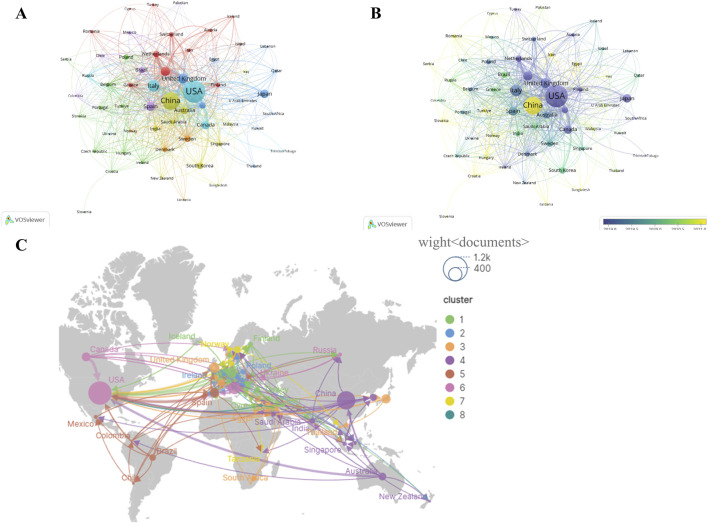
International collaboration network in T2DM and aging research. **(A)** Global country-level collaboration network generated using VOSviewer. Node size represents publication output, and link thickness reflects collaboration strength. **(B)** Temporal evolution of international research collaborations, visualized using VOSviewer. **(C)** Geographical distribution of international collaborations, visualized using SCImago Graphica.

Co-authorship analysis using VOSviewer grouped participating countries into eight distinct clusters (Figure 3A). The United States and China occupied central positions within the collaboration network and maintained extensive collaborative links with multiple countries. A collaboration cluster centered on the United States mainly included Canada, Italy, and Russia, whereas a second cluster centered on China comprised Australia, South Korea, Saudi Arabia, and India.

Figure 3B illustrates the temporal evolution of international collaborations. In the early stage, collaborations were primarily concentrated among the United States, Australia, France, Germany, and the Netherlands. After 2020, the number of participating countries increased, with expanded collaborative links observed for China, South Korea, and Brazil, suggesting a progressive expansion of the global collaboration network. Figure 3C further depicts intercontinental collaboration patterns, highlighting cross-continental cooperative connections.

### 3.3 Analysis of influential authors and co-citation networks

According to Table 2, Olivieri F ranked first in H-index (20), G-index (25), and M-index (1.25), with 25 publications in total. Prattichizzo F, Bonfigli AR, and Huang ES each authored 16 publications, reflecting consistent research productivity in this field. Among them, Prattichizzo F exhibited a relatively higher M-index, indicating stronger recent citation impact. Although Ferrucci L had a slightly lower H-index, he ranked first in total citations (TC = 2,747), indicating substantial academic influence. Overall, authors affiliated with institutions in Italy and the United States were prominently represented among the most influential contributors.

**TABLE 2:** Author impact metrics for T2DM and aging research (2019–2025).

Rank	Author	H-index	G-index	M-index	TC	NP	PY_start	Institution
1	Olivieri F	20	25	1.25	1,559	25	2011	Università politecnica delle Marche, Italy
2	Prattichizzo F	15	16	1.15	1,282	16	2014	IRCCS MultiMedica, Milan, Italy
3	Kirkland JL	13	13	1.00	2,066	13	2014	Mayo clinic, USA
4	Bonfigli AR	12	16	0.75	707	16	2011	INRCA IRCCS, Italy
5	Ferrucci L	12	13	0.75	2,747	13	2011	National institute on aging (NIA), NIH, USA
6	Giuliani A	12	14	1.00	889	14	2015	Università politecnica delle Marche, Italy
7	Procopio AD	12	12	0.75	1,145	12	2011	Università politecnica delle Marche, Italy
8	Huang ES	11	16	0.69	1,815	16	2011	University of Chicago, USA
9	Karter AJ	11	15	0.69	1,822	15	2011	Kaiser permanente Northern California, USA
10	Testa R	11	12	0.69	920	12	2011	INRCA IRCCS, Italy

The G-index, an enhancement of the H-index, assigns greater weight to highly cited articles. The M-index, calculated as the H-index divided by the number of years since the author’s first publication, enables comparison of academic productivity across career stages. NP, denotes the total number of publications, while PY_start indicates the year of the author’s first publication. Author names with potential duplicates (e.g., Chinese authors) were manually checked to ensure accuracy. Data were analyzed using *bibliometrix*.

**FIGURE 4:**
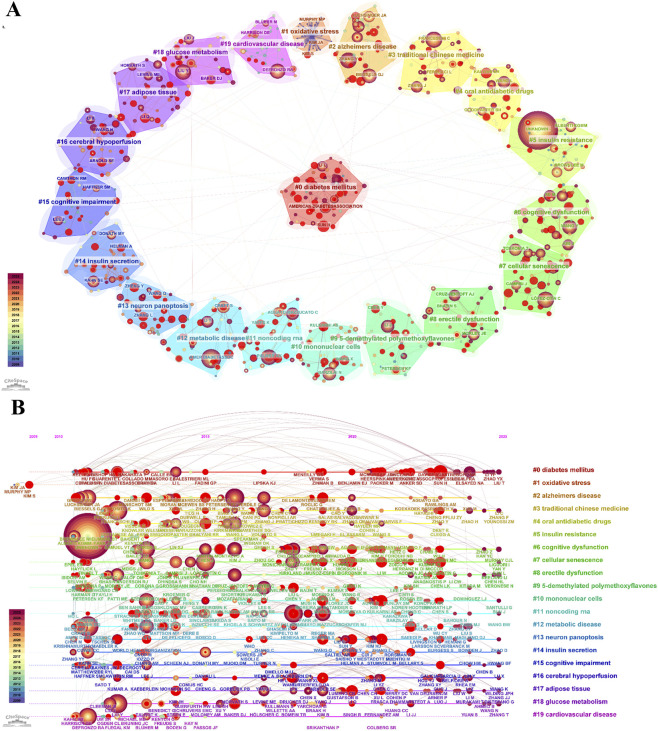
Author co-citation analysis in T2DM and aging research. **(A)** Cluster visualization of co-cited authors. Node size is proportional to total citation frequency; links indicate co-citation relationships, and link thickness reflects strength. Different colors represent distinct clusters, and red rings highlight authors with citation bursts. **(B)** Timeline view of co-citation evolution. The horizontal axis indicates the year of an author’s first citation; node size corresponds to total citation frequency, and the color gradient shows temporal progression.

Author co-citation network analysis revealed the structural characteristics of the knowledge base in this field (Figure 4A). The network comprised 1,003 co-cited authors and demonstrated a modularity value (Q = 0.8616, >0.3) and a mean silhouette score (S = 0.9381, >0.5), indicating a well-defined and robust clustering structure. A total of 20 clusters were identified, with the three largest labeled as #0 diabetes mellitus, #1 oxidative stress, and #2 Alzheimer’s disease.

From a temporal perspective, several clusters exhibited relatively recent temporal centroids. The mean publication year was 2019 for cluster #7 cellular senescence, cluster #10 mononuclear cells, and cluster #11 noncoding RNA, whereas cluster #17 adipose tissue had a mean year of 2021, indicating emerging research activity in these topics.

Timeline visualization of the co-citation network (Figure 4B) illustrates the temporal distribution of influential authors within the knowledge structure of this field. In the early phase (around 2011), authors such as Franceschi C, Biessels GJ, and Ferrucci L appeared prominently in the co-citation network. Around 2016, authors including López-Otín C, Palmer AK, and Aguayo-Mazzucato C became increasingly prominent in the network. By approximately 2020, Kulkarni AS, Elsayed NA, Saeedi P, and Ogrodnik M appeared more frequently in the co-citation network, reflecting their growing influence in recent research. Burst detection analysis further showed that Wild S (16.62), Petersen KF (14.78), and López-Otín C (14.06) exhibited the strongest citation bursts, indicating rapid increases in co-citation frequency during specific periods.

### 3.4 Institutional analysis

**FIGURE 5:**
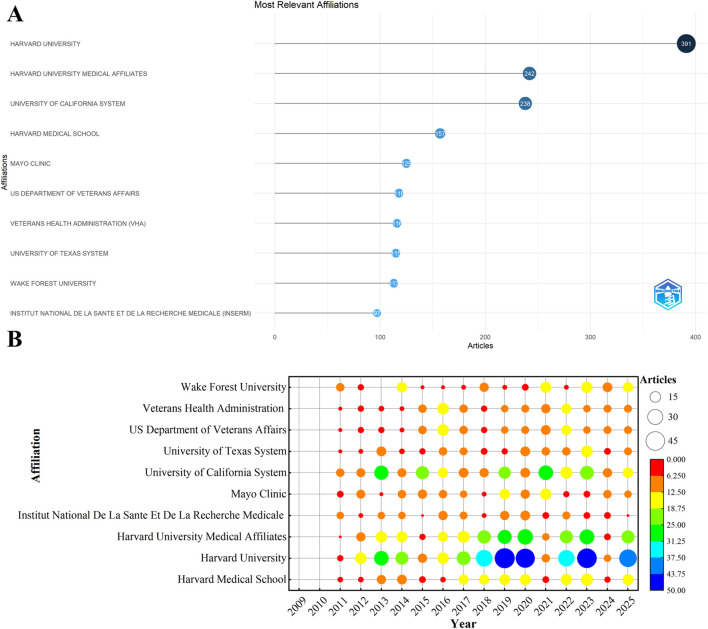
Institutional analysis. **(A)** Top 10 institutions by publication output. **(B)** Annual publication trends of the top 10 most productive institutions.

Harvard University, Harvard University Medical Affiliates, and the University of California System ranked as the top three institutions by publication output (Figure 5A). The bubble chart in Figure 5B indicates an overall upward trend in annual publications among the top 10 institutions, with elevated publication intensity observed in 2019, 2020, and 2023.

**FIGURE 6:**
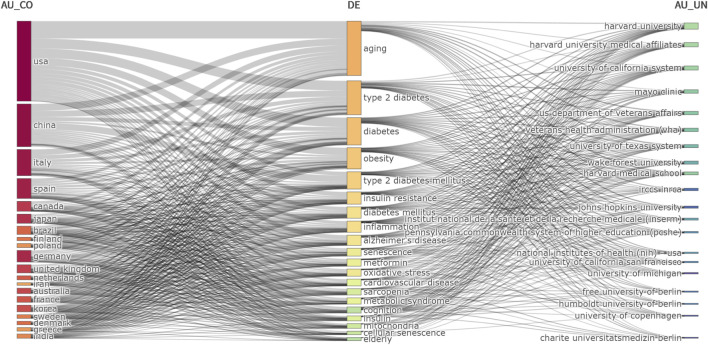
Country-keyword-institution network in T2DM and aging research.

The country–keyword–institution association network (Figure 6) shows that U.S. institutions exhibited high structural centrality, forming dense connections with core research themes including “aging,” “T2DM,” “senescence,” and “obesity.” Harvard University and the University of California System were positioned as central hubs in the network, demonstrating strong link strength across major research domains, including “aging,” “T2DM,” “insulin resistance,” “inflammation,” and “Alzheimer’s disease.”

### 3.5 Analysis of journals

**FIGURE 7:**
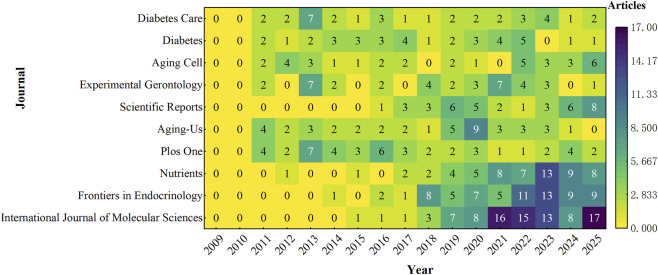
Annual publication heatmap of the top 10 journals in T2DM and aging research.

Figure 7 illustrates the publication distribution and temporal trends of major journals contributing to this field. *International Journal of Molecular Sciences* ranked first with 90 publications, followed by *Frontiers in Endocrinology* (71 publications) and *Nutrients* (60 publications). In contrast, traditional metabolism-focused journals such as *Diabetes Care* (35 publications) and *Diabetes* (35 publications) exhibited relatively stable publication output.

In terms of academic impact (Table 3), *Diabetes Care* had the highest average citations per article (106.91). *Aging-US* (94.79), *Aging Cell* (75.83), and *Ageing Research Reviews* (78.68) also demonstrated strong citation performance, constituting the core group of highly cited journals in this field.

**TABLE 3:** Journal impact metrics in T2DM and aging research (2019–2025).

Rank	Journal	H-index	G-index	M-index	TC	Average citation	NP	PY_start	If (2025)
1	International journal of molecular sciences	34	55	2.83	3,308	36.76	90	2015	4.9
2	Frontiers in endocrinology	27	55	2.08	3,155	44.44	71	2014	4.6
3	Diabetes care	26	35	1.63	3,742	106.91	35	2011	16.6
4	Aging-US	25	42	1.56	3,981	94.79	42	2011	3.9†
5	Diabetes	24	35	1.50	2,235	63.86	35	2011	7.5
6	PLOS ONE	23	39	1.44	1,597	34.72	46	2011	2.6
7	Ageing research reviews	22	34	1.47	2,675	78.68	34	2012	12.4
8	Aging cell	20	35	1.25	2,654	75.83	35	2011	7.1
9	Journal of clinical endocrinology & metabolism	20	32	1.25	1,511	47.22	32	2011	5.1
10	Nutrients	20	39	1.33	1,585	26.42	60	2012	5.0

Journal Impact Factor (IF) and JCR, Quartile data are from the 2025 Journal Citation Reports (Clarivate). Aging-US, 2025 metrics were unavailable; 2024 values are shown. Data were analyzed using *bibliometrix*.

**FIGURE 8:**
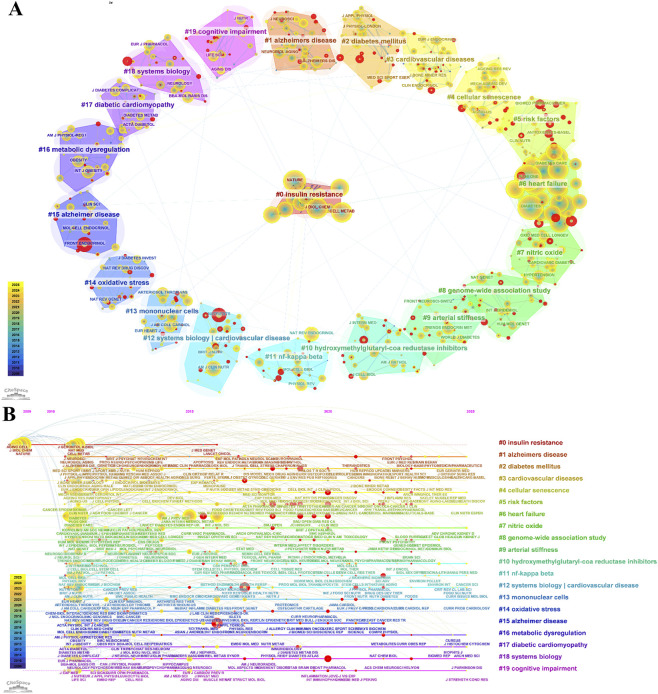
Journal co-citation analysis in T2DM and aging research. **(A)** Cluster visualization. **(B)** Timeline view of journal co-citation clusters.

Journal co-citation analysis revealed that a vascular complication–centered knowledge structure formed a key component of the co-citation network (Figure 8A). Cluster #3 cardiovascular disease served as the core cluster, showing strong co-citation linkages with Cluster #6 heart failure, Cluster #9 arterial stiffness, and Cluster #17 diabetic cardiomyopathy.

In terms of co-citation frequency, *Diabetes Care*, *PLOS ONE*, and *Diabetes* ranked among the top three journals with 1,709, 1,654, and 1,625 co-citations, respectively, all belonging to Cluster #6, indicating the structural prominence of this cluster. Additionally, *Frontiers in Endocrinology* within Cluster #15 Alzheimer’s disease exhibited the highest burst strength (45.56) (Figure 8B), reflecting a pronounced recent citation surge in this area.

**FIGURE 9:**
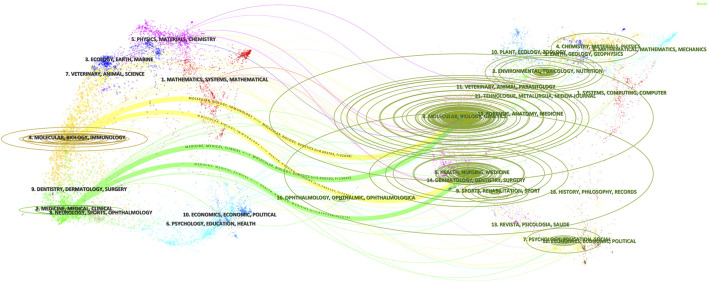
Dual-map overlay of journals in T2DM and aging research. The left clusters represent citing journals (research frontiers), whereas the right clusters represent cited journals (knowledge bases). Curved lines indicate citation pathways, with line thickness reflecting the strength of knowledge flow.

The dual-map overlay of journals (Figure 9) further delineated clear interdisciplinary knowledge flow pathways. Research outputs primarily originated from the domains of “Molecular, Biology & Genetics” and “Health, Nursing, Medicine,” and were directed along the yellow trajectory toward “Molecular, Biology, Immunology” journals and along the green trajectory toward “Medicine, Medical, Clinical” journals, illustrating a directional knowledge flow from basic research to clinical domains.

### 3.6 Analysis of co-cited references

**FIGURE 10:**
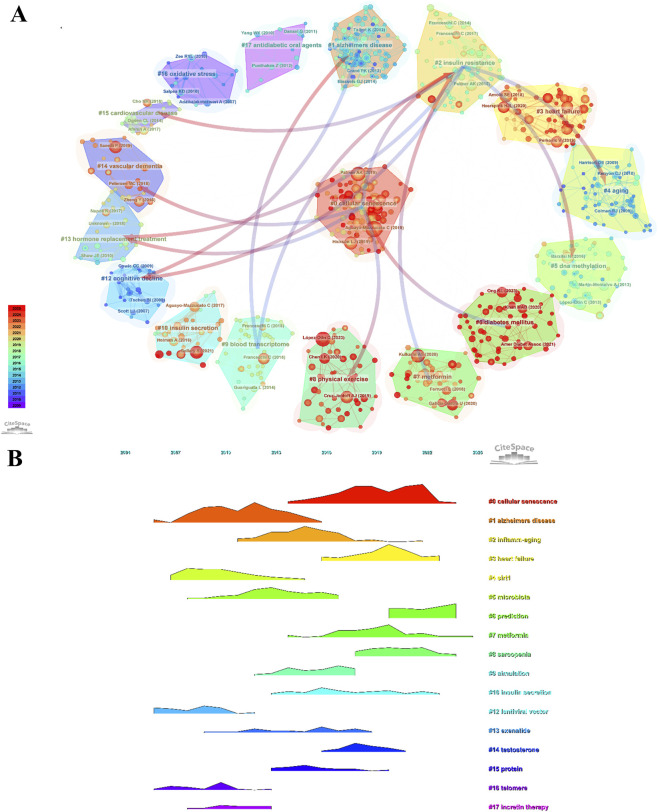
Co-citation analysis of references related to T2DM and aging. **(A)** Cluster visualization of co-cited references. **(B)** Cluster distribution of co-cited references.

The co-citation network exhibited a modularity (Q) of 0.8323 and a mean silhouette score (S) of 0.9262 (Figure 10A), indicating a robust and well-defined clustering structure. Three major clusters were identified: Cluster #0 cellular senescence (113 references; mean year = 2019), Cluster #1 Alzheimer’s disease (110 references; mean year = 2011), and Cluster #2 insulin resistance (68 references; mean year = 2014) (Figure 10B).

The representative reference of Cluster #0, published in *Cell Metabolism*, reported an association between β-cell senescence and metabolic dysfunction and investigated the effects of senescent cell clearance on disease phenotypes (Aguayo-Mazzucato et al., 2019). The key reference of Cluster #1, a cohort study published in *The New England Journal of Medicine*, analyzed the relationship between glycemic levels and dementia risk (Crane et al., 2013). The core reference of Cluster #2, entitled “Cellular senescence in type 2 diabetes: a therapeutic opportunity,” focused on the role of cellular senescence in the development and progression of T2DM (Palmer et al., 2015).

**FIGURE 11:**
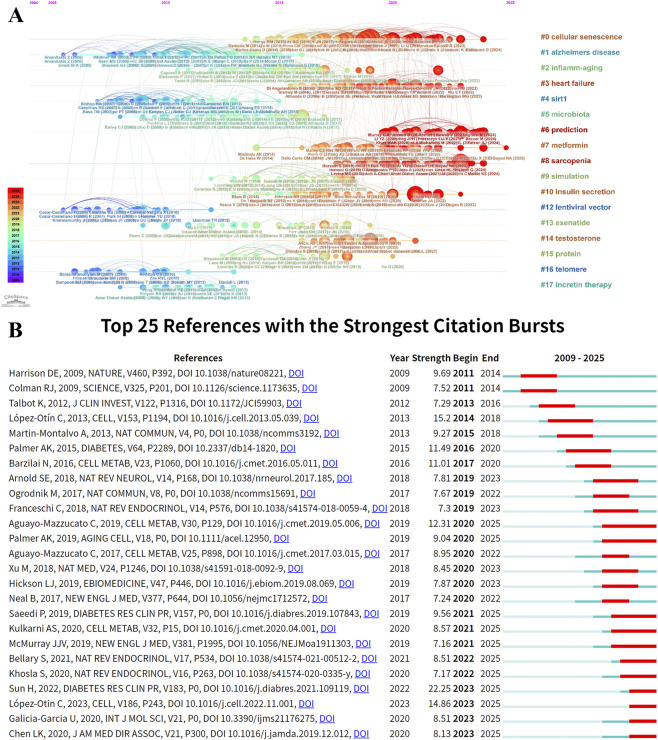
Citation burst analysis of co-cited references related to T2DM and aging. **(A)** Timeline view of co-cited reference clusters. **(B)** Top 25 references with the strongest citation bursts.

The timeline visualization (Figure 11A) indicates an initial concentration on epidemiological associations, followed by increasing attention to molecular and cellular mechanisms, and more recently to integrative frameworks centered on cellular senescence.

According to Table 4, the most frequently cited references were “Acceleration of β cell aging determines diabetes and senolysis improves disease outcomes” (n = 71) and “Cellular senescence in type 2 diabetes: a therapeutic opportunity” (n = 71), which constituted the core nodes of Cluster #0 and Cluster #2, respectively (Palmer et al., 2015; Aguayo-Mazzucato et al., 2019). The third-ranked reference was “Metformin improves healthspan and lifespan in mice” (n = 60), which investigated the effects of metformin on healthspan, lifespan, and metabolic homeostasis in experimental models (Martin-Montalvo et al., 2013).

**TABLE 4:** Top 10 publications ranked by local citations in T2DM and aging research (2019–2025).

Rank	Title	Journal	If (2025)	First author	Year	Local citations	Global citations	LC/GC ratio (%)
1	Acceleration of β cell aging determines diabetes and senolysis improves disease outcomes	Cell metabolism	30.9	Aguayo-mazzucato C	2019	71	355	20
2	Cellular senescence in type 2 diabetes: a Therapeutic opportunity	Diabetes	7.5	Palmer AK	2015	71	329	21.58
3	Metformin improves healthspan and lifespan in mice	Nature communications	15.7	Martin-montalvo A	2013	60	1125	5.33
4	Targeting senescent cells alleviates obesity-induced metabolic dysfunction	Aging cell	7.1	Palmer AK	2019	47	492	9.55
5	β cell aging markers have heterogeneous distribution and are induced by insulin resistance	Cell metabolism	30.9	Aguayo-mazzucato C	2017	37	174	21.26
6	Cellular senescence: At the nexus between ageing and diabetes	Diabetologia	10.2	Palmer AK	2019	34	263	12.93
7	Metformin retards aging in *C. elegans* by altering microbial folate and methionine metabolism	Cell	42.5	Cabreiro F	2013	32	786	4.07
8	The role of cellular senescence in ageing and endocrine disease	Nature reviews endocrinology	40	Khosla S	2020	31	404	7.67
9	Type 2 diabetes and the aging pancreatic beta cell	Aging-US	3.9†	Gunasekaran U	2011	25	154	16.23
10	Obesity and type-2 diabetes as inducers of premature cellular senescence and ageing	Biogerontology	4.1	Burton DGA	2018	23	142	16.20

Publications were ranked according to local citations within the retrieved dataset. IF, data were obtained from the 2025 Journal Citation Reports (Clarivate). Aging-US, 2025 metrics were unavailable; 2024 values are shown. Data were analyzed using *bibliometrix*.

Burst detection analysis showed that “IDF Diabetes Atlas: global, regional and country-level diabetes prevalence estimates for 2021 and projections for 2045” exhibited the highest burst strength (22.25), followed by “The hallmarks of aging” (15.20) (Table 5) (López-Otín et al., 2013; Sun et al., 2022). In addition, “Hallmarks of aging: an expanding universe” demonstrated sustained burst intensity in recent years, indicating increasing citation attention (Figure 11B) (López-Otín et al., 2023).

**TABLE 5:** Top 10 references with the strongest citation bursts in T2DM and aging research (2019–2025).

Rank	Title	Bursts	Journal	If (2025)	First author	Year
1	IDF diabetes atlas: global, regional and country-level diabetes prevalence estimates for 2021 and projections for 2045	22.25	Diabetes research and clinical practice	7.4	Sun H	2022
2	The hallmarks of aging	15.20	Cell	42.5	López-Otín C	2013
3	Hallmarks of aging: An expanding universe	14.86	Cell	42.5	López-Otín C	2023
4	Acceleration of β cell aging determines diabetes and senolysis improves disease outcomes	12.31	Cell metabolism	30.9	Aguayo-mazzucato C	2019
5	Cellular senescence in type 2 diabetes: a Therapeutic opportunity	11.49	Diabetes	7.5	Palmer AK	2015
6	Metformin as a tool to target aging	11.01	Cell metabolism	30.9	Barzilai N	2016
7	Rapamycin fed late in life extends lifespan in genetically heterogeneous mice	9.69	Nature	48.5	Harrison DE	2009
8	Global and regional diabetes prevalence estimates for 2019 and projections for 2030 and 2045: Results from the international diabetes Federation diabetes atlas, 9th edition	9.56	Diabetes research and clinical practice	7.4	Saeedi P	2019
9	Metformin improves healthspan and lifespan in mice	9.27	Nature communications	15.7	Martin-montalvo A	2013
10	Targeting senescent cells alleviates obesity-induced metabolic dysfunction	9.04	Aging cell	7.1	Palmer AK	2019

Bursts indicate references that received a sudden increase in citations within the dataset. Citation bursts were detected using CiteSpace software. IF, data were obtained from the 2025 Journal Citation Reports (Clarivate).

### 3.7 Keyword analysis

Based on keyword co-occurrence analysis, a knowledge structure map of T2DM and aging research was constructed. The most frequently occurring terms were “type 2 diabetes mellitus” (n = 945), “insulin resistance” (n = 583), and “oxidative stress” (n = 423) (Table 6).

**TABLE 6:** Top 20 keywords by frequency in T2DM and aging research (2009–2025).

Rank	Keywords	Frequency	Year	Rank	Keywords	Frequency	Year
1	Type 2 diabetes mellitus	945	2011	11	Mellitus	192	2011
2	Insulin resistance	583	2011	12	Disease	189	2011
3	Oxidative stress	423	2011	13	Expression	189	2011
4	Risk	355	2011	14	Risk factors	180	2011
5	Alzheimer’s disease	273	2011	15	Older adults	177	2011
6	Diabetes mellitus	251	2011	16	Prevalence	176	2011
7	Metabolic syndrome	237	2011	17	Skeletal muscle	162	2011
8	Association	230	2011	18	Inflammation	161	2011
9	Cardiovascular disease	227	2011	19	Adipose tissue	158	2011
10	Obesity	201	2011	20	Age	155	2011

Keywords were extracted and analyzed using CiteSpace software. “Frequency” indicates the number of publications in which the keyword appeared. “Year” indicates the earliest year the keyword appeared in the dataset.

**FIGURE 12:**
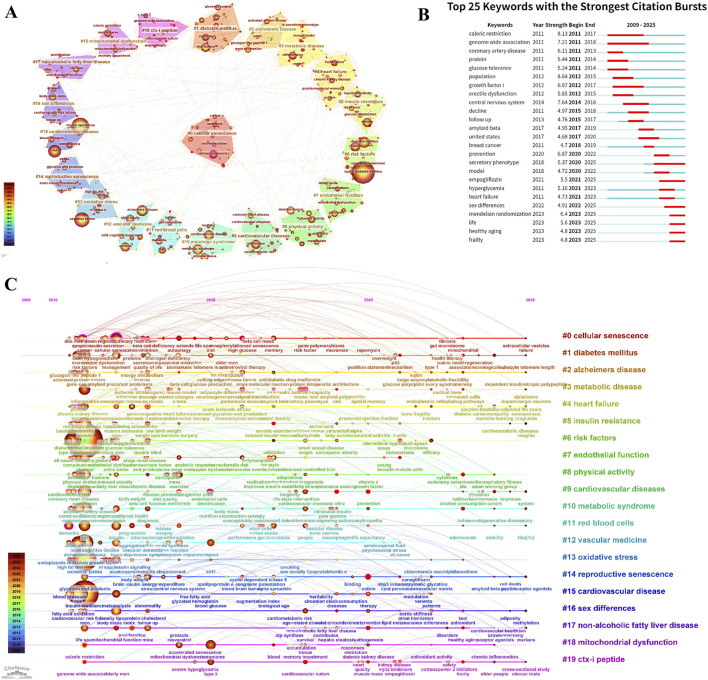
Keyword analysis related to T2DM and aging. **(A)** Cluster visualization of keywords. **(B)** Top 25 keywords with the strongest citation bursts. **(C)** Timeline view of keywords.

Cluster analysis yielded 20 major clusters (Figure 12A). Cluster #0 cellular senescence was the largest cluster (size = 39), followed by Cluster #1 diabetes mellitus (size = 36) and Cluster #2 Alzheimer’s disease (size = 32). Representative keywords within these clusters included “cellular senescence,” “apoptosis,” “DNA methylation,” “risk factors,” “glycemic control,” and “glucagon-like peptide-1.”

The timeline visualization illustrated the temporal evolution of research priorities (Figure 12C). From 2010 to 2015, research primarily focused on themes such as “cellular senescence,” “inflammation,” “oxidative stress,” “T2DM,” “Alzheimer’s disease,” “metabolic syndrome,” and “insulin resistance.” Between 2016 and 2020, research attention expanded to topics including “SASP,” “stem cells,” “cardiovascular diseases,” “Parkinson’s disease,” and “gut microbiota.” Since 2021, studies have increasingly emphasized mechanistic processes, including “homeostasis,” “NLRP3 inflammasome,” “T cells,” “apoptosis,” and “fibrosis.”

Keyword burst detection highlighted emerging research fronts (Table 7). The strongest burst terms were “population” (8.64), “caloric restriction” (8.13), and “central nervous system” (7.64). Additionally, “Mendelian randomization” (6.40), “empagliflozin” (5.50), and “secretory phenotype” (5.37) exhibited recent burst activity, suggesting continued scholarly interest and potential future research directions (Figure 12B).

**TABLE 7:** Top 20 keywords with the strongest citation bursts in T2DM and aging research (2009–2025).

Rank	Keywords	Burst	Burst begin	Burst end	Frequency	Year	Rank	Keywords	Burst	Burst begin	Burst end	Frequency	Year
1	Population	8.64	2012	2015	67	2012	11	Empagliflozin	5.50	2021	2025	17	2021
2	Caloric restriction	8.13	2011	2017	36	2011	12	Protein	5.44	2011	2014	51	2011
3	Central nervous system	7.64	2014	2018	22	2014	13	Secretory phenotype	5.37	2020	2025	42	2020
4	Genome-wide association	7.21	2011	2018	40	2011	14	Glucose tolerance	5.24	2011	2014	66	2011
5	Growth factor I	6.97	2012	2017	34	2012	15	Hyperglycemia	5.16	2021	2023	21	2021
6	Prevention	6.87	2020	2022	16	2020	16	Decline	4.97	2015	2018	40	2015
7	Mendelian randomization	6.40	2023	2025	16	2023	17	Amyloid-β	4.95	2017	2019	17	2017
8	Coronary artery disease	6.11	2011	2013	21	2011	18	sex differences	4.91	2022	2025	12	2022
9	Erectile dysfunction	5.85	2012	2015	11	2012	19	Frailty	4.80	2023	2025	12	2023
10	life	5.60	2023	2025	14	2023	20	Healthy aging	4.80	2023	2025	12	2023

Citation burst keywords were identified using CiteSpace. “Burst” indicates the strength of citation increase, while “Burst Begin” and “Burst End” denote the duration of the burst. “Year” represents the first occurrence of the keyword in the dataset (2009–2025).

Centrality analysis identified key hubs within the knowledge network (Table 8). “Cellular senescence” (centrality = 0.22), “DNA methylation” (0.17), and “C-reactive protein” (0.17) ranked highest, underscoring their pivotal roles in connecting different research themes.

**TABLE 8:** Top 20 keywords by betweenness centrality in T2DM and aging research (2009–2025).

Rank	Keywords	Centrality	Frequency	Year	Rank	Keywords	Centrality	Frequency	Year
1	cellular senescence	0.22	135	2012	11	Endothelial cells	0.11	24	2015
2	C-reactive protein	0.17	39	2011	12	Glucose metabolism	0.10	60	2011
3	DNA methylation	0.17	59	2011	13	Dementia	0.10	98	2011
4	Bone mineral density	0.16	43	2011	14	Complications	0.10	67	2011
5	β cells	0.15	28	2013	15	Activated protein kinase	0.09	67	2011
6	Insulin sensitivity	0.14	76	2011	16	Cardiovascular risk factors	0.09	36	2011
7	Apoptosis	0.12	62	2011	17	Activation	0.09	103	2011
8	Chronic disease	0.12	18	2013	18	Glucagon-like peptide-1	0.09	40	2011
9	Health	0.11	151	2011	19	Cardiovascular risk	0.09	32	2011
10	Endothelial dysfunction	0.11	43	2012	20	Coronary artery disease	0.08	21	2011

Centrality measures the importance of a keyword in connecting different clusters within the network, indicating its influence in the field. Keywords were extracted and analyzed using CiteSpace.

## 4 Discussion

### 4.1 Overall trends and global collaboration patterns

Since 2016, annual publication output in this field has shown a sustained and pronounced upward trajectory, peaking in 2025 (n = 344). The fitted curve (R^2^ = 0.970) suggests a highly consistent growth trajectory. This growth phase coincides with major conceptual advances in aging biology. In 2015, seminal studies by Kirkland JL and colleagues demonstrated that selective elimination of senescent cells (senolytics) can extend healthspan in experimental models, improve cardiovascular function, and enhance physical performance (Zhu et al., 2015). Concurrently, accelerating global population aging and the escalating burden of diabetes have imposed substantial public health challenges, further directing research investment toward this interdisciplinary domain (Sun et al., 2022).

Disciplinary distribution analysis revealed a dual-dominant framework composed of Endocrinology & Metabolism and Geriatrics & Gerontology, underscoring the deep integration of metabolic disease research with aging biology. The dual-map overlay further indicated a knowledge-flow transition from “Molecular, Biology & Genetics” to “Medicine, Medical, Clinical,” reflecting a paradigm shift from mechanistic exploration toward clinical translation.

The global collaboration network displays a dual-core collaboration structure centered on the United States and China. The United States remains dominant in publication volume, network centrality, and the breadth of international collaborations, supported by multiple institutional hubs. China ranks second in output and has demonstrated a marked expansion in international collaborative linkages, signaling rapidly increasing academic engagement. Nevertheless, in terms of multinational collaboration proportion and the structure of highly cited outputs, European and North American countries continue to hold advantages in high-impact original research and collaboration quality. These findings indicate that emerging research systems, while expanding in scale, should further strengthen originality and deepen cross-national collaboration.

At the journal level, a distinct stratification of roles is evident. *International Journal of Molecular Sciences* and *Frontiers in Endocrinology* lead in publication volume, whereas *Diabetes Care*, *Aging Cell*, and *Ageing Research Reviews* rank highest in average citations per article. Among researchers, Olivieri F, Prattichizzo F, and Huang ES are the most prolific contributors, while López-Otín C, Palmer AK, and Kirkland JL have exerted lasting influence through landmark contributions that have shaped the field’s trajectory (López-Otín et al., 2013; Zhu et al., 2015; Palmer et al., 2019).

### 4.2 Research hotspots and frontier evolution

Co-citation and keyword analyses collectively delineate the paradigm shift within this research domain. The co-citation network identifies Cluster #0 cellular senescence (mean year = 2019) as the largest and most active cluster, highlighting cellular senescence as the central mechanistic framework linking T2DM with systemic aging. β-cell senescence is strongly associated with islet dysfunction, while SASP-driven chronic inflammation aggravates insulin resistance. Importantly, experimental clearance of senescent cells ameliorates metabolic phenotypes, reinforcing the causal relevance of senescence in metabolic decline. Cluster #1 Alzheimer’s disease and Cluster #2 insulin resistance represent neurodegenerative complications and metabolic dysregulation, respectively, underscoring the systemic and cross-organ nature of T2DM-related aging processes.

Timeline analysis further illustrates the dynamic reorientation of research priorities. Early investigations (2010–2015) centered on classical pathogenic mechanisms, including oxidative stress and inflammation. The intermediate phase (2016–2020) expanded toward systemic-level interactions, encompassing SASP, gut microbiota, and cardiovascular diseases. In the most recent stage (2021–2025), attention has shifted toward refined molecular regulatory circuits—such as the NLRP3 inflammasome, T cells, and homeostatic signaling networks—signaling a transition toward integrated, multi-pathway mechanistic models.

Burst keyword detection further highlights emerging research frontiers. Persistent bursts of “population” and “caloric restriction” confirm the sustained importance of epidemiological research and metabolic intervention strategies. The emergence of “Mendelian randomization” and “empagliflozin” reflects the integration of causal inference methodologies and novel glucose-lowering therapies into aging-oriented evaluation frameworks. Continued attention to research aligned with the “Hallmarks of Aging” further positions classical aging theory as an overarching conceptual framework for mechanistically interpreting T2DM progression.

### 4.3 The relationship between T2DM and cellular senescence

Our bibliometric analysis identifies cellular senescence as a central hub connecting T2DM and aging research networks. As shown in Figures 10, 12, this topic represents both the largest research cluster and the most central keyword (Table 8). These bibliometric findings align with accumulating molecular evidence, collectively establishing cellular senescence as fundamental to T2DM pathophysiology.

#### 4.3.1 A bidirectional feedback loop: mutually reinforcing mechanisms of T2DM and senescence

A key conceptual advance in this field is the establishment of a bidirectional vicious cycle in which T2DM and cellular senescence mutually reinforce each other. This framework provides a unified perspective for understanding the progressive deterioration of T2DM and its association with systemic aging, marking a shift from linear causality to dynamic network interactions. Multiple lines of evidence support this paradigm.

Clinical observations reveal that increased senescent cell burden precedes classic manifestations such as hyperglycemia in T2DM-susceptible individuals (Gustafson et al., 2019), indicating that senescent cell accumulation represents an early driver rather than a late consequence of T2DM pathogenesis. Mechanistically, accumulated senescent cells disrupt metabolic homeostasis across multiple organs through the SASP. In adipose tissue, senescent cells sustain chronic low-grade inflammation through continuous release of interleukin-6, monocyte chemoattractant protein-1, and plasminogen activator inhibitor-1 (Kim et al., 2006; Freund et al., 2010), ultimately promoting systemic insulin resistance (Esser et al., 2014). Senescent adipocytes further recruit and polarize monocytes toward M1 macrophages within the SASP microenvironment, establishing an inflammatory amplification loop that accelerates T2DM progression (Xu et al., 2015).

In pancreatic islets, senescent β-cells exhibit both intrinsic dysfunction with impaired insulin secretion and capacity to induce bystander senescence in healthy β-cells through paracrine release of interleukin-1β, interleukin-6, and tumor necrosis factor-α (Aguayo-Mazzucato et al., 2017; 2019; Aguayo-Mazzucato, 2020). These inflammatory mediators promote immune infiltration into islets, exacerbating local inflammation and leading to progressive β-cell loss (Donath et al., 2009; Bramer et al., 2018; Chan et al., 2019).

Conversely, T2DM-associated metabolic disturbances create a potent pro-senescence microenvironment (Iwasaki et al., 2023). Persistent hyperglycemia induces cellular senescence through multiple interconnected mechanisms: (1) oxidative stress-mediated DNA damage and telomere dysfunction (Pham-Huy et al., 2008; Coluzzi et al., 2014), (2) the formation of advanced glycation end products and receptor for advanced glycation end products activation driving inflammation (Peppa et al., 2003; Liu et al., 2014), (3) mitochondrial dysfunction increasing reactive oxygen species production (Pinti et al., 2019; Wang et al., 2019), (4) endoplasmic reticulum stress induction (He et al., 2020). These stressors converge on p53/p21^CIP1^ and p16^INK4a^/Rb pathways, upregulating cell cycle inhibitors to irreversibly establish senescence (Ohanna et al., 2011; Dookun et al., 2022).

*In vitro* studies confirm high glucose directly upregulates p16^INK4a^ and promotes SASP secretion in β-cells (Marino et al., 2022). Beyond hyperglycemia, T2DM-associated lipotoxicity promotes senescence via p53 and p38 mitogen-activated protein kinase activation (Yamakoshi et al., 2009; Shimizu et al., 2015), while growth hormone/insulin-like growth factor-1 axis dysregulation induces premature aging through p53-dependent mechanisms in insulin-resistant states (Serrano et al., 1997; Collado et al., 2005; Fuhrmann-Stroissnigg et al., 2017). Crucially, diabetes-generated senescent cells propagate aging through SASP-mediated bystander effects, establishing a self-reinforcing feedback loop that accelerates tissue aging and metabolic dysfunction (Aguayo-Mazzucato et al., 2019).

#### 4.3.2 The systemic aging nexus: senescence at the core of T2DM comorbidities

Bibliometric analysis revealed that clusters such as “Alzheimer’s disease,” “cardiovascular disease,” “erectile dysfunction,” and “cognitive impairment” collectively form a T2DM-centered comorbidity network. This pattern suggests that these age-related conditions may share a common pathological basis beyond traditional metabolic pathways, with systemic aging—driven by senescent cell accumulation and chronic inflammation—serving as the central mechanistic hub (Khosla et al., 2020).

Within this framework, the systemic aging process mediates multi-organ pathology through distinct mechanisms. In the nervous system, persistent hyperglycemia induces endoplasmic reticulum stress, contributing to Aβ accumulation and tau hyperphosphorylation, thereby significantly increasing the risk of Alzheimer’s disease (He et al., 2020). Concurrently, the diabetic environment exacerbates oxidative stress and neuroinflammation by suppressing Nrf2-mediated antioxidant pathways, collectively promoting cognitive decline (Zhao et al., 2018; Ryder et al., 2020).

In the cardiovascular system, the diabetic microenvironment impairs endothelial function through mechanisms including lipotoxicity while also promoting the secretion of pro-thrombotic SASP factors such as plasminogen activator inhibitor-1 from senescent cells, consequently accelerating cardiac progenitor cell senescence, impairing regenerative potential, and increasing atherosclerotic plaque instability (Rota et al., 2006; Minamino and Komuro, 2007). In the skeletal system, increased advanced glycation end products resulting from diabetes or aging elevate bone fragility by inducing type I collagen cross-linking, thereby increasing fracture risk (Yamamoto and Sugimoto, 2016). Clinical studies demonstrate positive correlations between p16/p21 expression in T cells and glycated hemoglobin levels in T2DM patients, with individuals exhibiting high p16 expression showing significantly reduced tibial cortical area, suggesting intrinsic connections between immunosenescence and abnormal bone metabolism (Hoong et al., 2025).

Senescent cell accumulation is prevalent in patients with T2DM. Compared to non-diabetic individuals, tissues including kidney, retina, and pancreatic islets exhibit significantly elevated expression of senescence markers such as senescence-associated β-galactosidase activity and p16^INK4a^ (Liu et al., 2014; Aguayo-Mazzucato et al., 2019; Crespo-Garcia et al., 2021). Targeted clearance of these senescent cells effectively alleviates various diabetes-associated tissue pathologies and functional decline (Crespo-Garcia et al., 2021).

#### 4.3.3 Expansion of therapeutic strategies: from glycemic control to aging-targeted interventions

With the growing recognition of aging as a key contributor to T2DM pathogenesis, therapeutic strategies are undergoing a strategic expansion. Within the established framework of glycemic control–centered management, interventions targeting aging-related biological mechanisms are increasingly investigated as complementary approaches with potential disease-modifying effects (Palmer et al., 2015).

Keyword analysis reveals a continuous increase in the burst strength of empagliflozin, reflecting growing research interest in the anti-aging potential of glucose-lowering drugs. Current therapeutic strategies targeting aging mechanisms in T2DM primarily advance along three major directions. The most representative approach employs senolytics to selectively induce apoptosis in senescent cells (Zhu et al., 2015). The combination of dasatinib and quercetin demonstrated significant efficacy in a clinical trial of patients with diabetic kidney disease, substantially reducing p16^INK4a+^ and p21^CIP1+^ cells in adipose tissue and skin within 3 days of treatment, alongside markedly decreased circulating SASP factors (Hickson et al., 2019). In obese diabetic mouse models, this regimen effectively improved glucose tolerance and insulin sensitivity by clearing senescent cells from white adipose tissue (Zhu et al., 2015).

Unlike senolytics, senomorphics aim to block detrimental SASP effects without eliminating senescent cells. Mechanistic target of rapamycin inhibitors (e.g., rapamycin) and Janus kinase inhibitors (e.g., ruxolitinib) demonstrate the capacity to suppress SASP production (Xu et al., 2015). Additionally, repurposing conventional medications reveals unique therapeutic value. Metformin has been confirmed to inhibit SASP production by interfering with NF-κB signaling, potentially representing a novel mechanism for its benefits against diabetes and complications (Moiseeva et al., 2013). Emerging research indicates that empagliflozin extends healthspan and delays hepatic aging in natural aging models through mechanisms involving downregulation of key senescence markers p16/p21, modulation of gut microbiota, and regulation of PI3K/AKT/P21 and AMPK/SIRT1/NF-κB signaling pathways (Long et al., 2024). For renal protection, it alleviates renal tubular epithelial cell senescence by activating the Six1/Wnt4 pathway and inhibiting NF-κB signaling (Chen et al., 2025), while also demonstrating close association with blocking the pro-inflammatory senescence axis formed by advanced glycation end products and their receptor for advanced glycation end products (Guvatova et al., 2025; Matsui et al., 2025).

Beyond these emerging therapeutic directions, a clearer translational framework is warranted to bridge mechanistic insights and clinical application. Based on the current research landscape, clinical translation may evolve through several sequential stages. First, continued mechanistic investigations are needed to clarify the causal contribution of cellular senescence to organ-specific and systemic complications of T2DM. Second, the development of reliable and quantifiable senescence-associated biomarkers may facilitate patient stratification and therapeutic monitoring. Third, well-designed early-phase clinical studies are essential to evaluate safety profiles and biological responsiveness of aging-targeted interventions. Ultimately, large-scale, long-term trials will be required to determine their clinical efficacy and integration within the established framework of glycemic control. Rather than replacing conventional metabolic management, aging-targeted strategies may function as complementary components within a multidimensional treatment paradigm.

### 4.4 Implications

Through a systematic analysis of 3,048 publications spanning nearly 17 years, this study presents a novel data-driven framework for mapping the dynamic knowledge structure of research at the interface of T2DM and aging. Bibliometric evidence clearly identifies cellular senescence as a central hub within this research network, offering a new theoretical perspective for understanding the systemic pathophysiology of T2DM.

Cluster analyses reveal stable comorbidity patterns, including Alzheimer’s disease, cardiovascular disease, and erectile dysfunction, providing macroscopic support for the hypothesis that T2DM represents a state of systemic accelerated aging. Research trajectories indicate a progression from early phenotypic characterization toward mechanistic exploration, with an expansion from single-organ studies to integrative network analyses. Concurrently, the emergence of senolytics, senomorphics, and drug repurposing underscores the field’s movement from mechanistic elucidation toward prospective therapeutic strategies. Collectively, these findings establish a logical link between basic research and clinical translation, offering novel theoretical support for refining long-term management frameworks for T2DM.

## 5 Limitations

This study has several limitations. First, the data were exclusively obtained from the Web of Science Core Collection. Although this database is widely recognized for its authority and standardization, reliance on a single source may introduce selection bias and potentially exclude relevant literature not indexed in this database. Second, bibliometric analyses are inherently dependent on published outputs and may not fully capture emerging research directions that remain in early developmental stages. Third, the present study primarily employed quantitative bibliometric methods and did not conduct a systematic content evaluation of key publications.

Future studies may address these limitations by integrating multiple databases, extending the observation period, and incorporating qualitative approaches, such as systematic reviews or expert consensus, to achieve a more comprehensive and in-depth understanding of this research field.

## 6 Conclusion

This study systematically delineates the knowledge structure and developmental trajectory at the intersection of T2DM and aging. Since 2016, the field has entered a phase of rapid expansion, forming an international collaborative network centered on the United States and China while demonstrating a clear transition from basic mechanistic investigations toward translational applications.

Bibliometric evidence identifies cellular senescence as a structurally central node within the T2DM–aging interaction network. Through SASP-mediated mechanisms, senescent cells contribute to multi-organ functional imbalance, thereby linking metabolic dysregulation with systemic aging processes. Stable clusters of multi-system comorbidities further support the conceptualization of T2DM as a condition characterized by systemically accelerated aging. Research priorities have gradually shifted from early emphasis on oxidative stress and inflammation toward deeper exploration of molecular pathways, including SASP signaling, the NLRP3 inflammasome, and epigenetic regulation.

Meanwhile, the potential anti-aging effects of senolytics, senomorphics, and certain glucose-lowering drugs have emerged as promising directions, offering complementary disease-modifying strategies for long-term T2DM management. Based on the current research landscape and evolving hotspots, future studies may further investigate tissue-specific regulation of core aging pathways and the systemic integration of multi-organ networks mediated by inflammatory and SASP signaling. In addition, advancing mechanistic validation, biomarker development, and phased clinical evaluation will be essential for facilitating the clinical translation of aging-targeted interventions. Within the foundational framework of glycemic control, these strategies hold the potential to complement existing therapies and contribute to more comprehensive long-term management of T2DM.
